# Discovery of induced point mutations in maize genes by TILLING

**DOI:** 10.1186/1471-2229-4-12

**Published:** 2004-07-28

**Authors:** Bradley J Till, Steven H Reynolds, Clifford Weil, Nathan Springer, Chris Burtner, Kim Young, Elisabeth Bowers, Christine A Codomo, Linda C Enns, Anthony R Odden, Elizabeth A Greene, Luca Comai, Steven Henikoff

**Affiliations:** 1 Basic Sciences Division, Fred Hutchinson Cancer Research Center, Seattle, Washington 98109, USA; 2 Department of Biology, University of Washington, Seattle, Washington 98195, USA; 3 Department of Agronomy, Purdue University, West Lafayette, Indiana 47907, USA; 4 Department of Plant Biology, University of Minnesota, St. Paul, Minnesota, 55108 USA

## Abstract

**Background:**

Going from a gene sequence to its function in the context of a whole organism requires a strategy for targeting mutations, referred to as reverse genetics. Reverse genetics is highly desirable in the modern genomics era; however, the most powerful methods are generally restricted to a few model organisms. Previously, we introduced a reverse-genetic strategy with the potential for general applicability to organisms that lack well-developed genetic tools. Our TILLING (Targeting Induced Local Lesions IN Genomes) method uses chemical mutagenesis followed by screening for single-base changes to discover induced mutations that alter protein function. TILLING was shown to be an effective reverse genetic strategy by the establishment of a high-throughput TILLING facility and the delivery of thousands of point mutations in hundreds of Arabidopsis genes to members of the plant biology community.

**Results:**

We demonstrate that high-throughput TILLING is applicable to maize, an important crop plant with a large genome but with limited reverse-genetic resources currently available. We screened pools of DNA samples for mutations in 1-kb segments from 11 different genes, obtaining 17 independent induced mutations from a population of 750 pollen-mutagenized maize plants. One of the genes targeted was the DMT102 chromomethylase gene, for which we obtained an allelic series of three missense mutations that are predicted to be strongly deleterious.

**Conclusions:**

Our findings indicate that TILLING is a broadly applicable and efficient reverse-genetic strategy. We are establishing a public TILLING service for maize modeled on the existing Arabidopsis TILLING Project.

## Background

Rapid progress being made in genome sequencing projects provides raw material for the potential understanding of gene function, so effective reverse genetic strategies are increasingly in demand [1]. Sequence information alone may be sufficient to consider a gene to be of interest, because sequence comparison tools that detect protein sequence similarity to previously studied genes often allow a related function to be inferred. Hypotheses concerning gene function that are generated in this way must be confirmed empirically. Experimental determination of gene function is desirable in other situations as well, for example, when a genetic interval has been associated with a phenotype of interest. In such cases, the functions of genes in an interval can be deduced from the phenotypes of induced mutations. Furthermore, the dissection of gene interactions often requires the availability of a range of allele types. However, most available methods for inferring function rely on techniques that produce a limited range of mutations, are labor-intensive or unreliable, or are limited to species in which special genetic tools have been developed [2]. Just as the discovery of induced mutations led to forward genetics, the introduction of rapid reverse genetic methods can have great impact.

Several general strategies have been used to obtain reduction-of-function or knockout mutations in model organisms, including insertional mutagenesis [3] and RNA suppression [4], which have been widely used in plants. Insertional mutagenesis is now largely an *in silico *procedure for Arabidopsis researchers, as searchable databases of flanking sequences from T-DNA and transposon insertions are available on-line [5]. RNA suppression currently requires considerable manual effort, but it has the potential of reducing expression of repeated genes, which are especially common in plants, including Arabidopsis. However, because these techniques rely either on Agrobacterium T-DNA vectors for transmission or on endogenous tagging systems, their usefulness as general reverse genetics methods is limited to very few plant species.

In maize, the majority of reverse genetic resources have exploited the endogenous *Mutator (Mu) *and *Activator (Ac) *transposon families. Mutator transposable elements are frequently present in high copy number and tend to insert in or near genes [6] thus providing mostly gene knock-out and potential loss-of-function alleles due to insertions in regulatory elements. Resources to identify *Mu *insertions in target genes include the Trait Utility System for Corn [7], the Maize Targeted Mutagenesis project [8], and the Photosynthetic Mutant Hunt [9]. Additionally, a modified *Mutator *element has been engineered to allow plasmid rescue of the element and flanking genomic DNA into *E. coli *[10,11] facilitating the sequencing of tagged genes and allowing the generation of an insertional database analogous to the T-DNA database currently available for Arabidopsis. *Activator *transposons are low copy number elements that, like *Mu, *preferentially insert in or near genes [12]. Large-scale isolation and sequencing of *Ac *elements for a reverse genetics database is advantageous, because single to low copy number insertions can be obtained.

Reverse genetic strategies based on induced mutations have the potential for general applicability. Two such methods have been described for plants. One is deletional mutagenesis using fast neutron bombardment, which appears to be an effective means of knocking out tandemly repeated genes [13]. Another is TILLING (Targeting Induced Local Lesions IN Genomes), in which treatment using traditional chemical mutagens causes point mutations that are then discovered in genes of interest using a sensitive method for single-nucleotide mutation detection [14]. TILLING can provide an allelic series that includes missense and knockout mutations. The utility of allelic series that has been demonstrated in traditional forward genetic studies makes TILLING an especially desirable reverse-genetic strategy as genomic sequences become increasingly available.

We have introduced a high-throughput screening method for TILLING based on the use of a mismatch-cleavage endonuclease, followed by fluorescent display of cleaved products on polyacrylamide electrophoretic gels using the LI-COR analyzer system [15]. We systematized this method and established a public TILLING facility for the general Arabidopsis community [16]. Our Arabidopsis TILLING Project (ATP) screened on average ~3000 ethylmethanesulfonate (EMS)-mutagenized M2 Arabidopsis plants per 1-kb gene fragment, which resulted in the delivery of >4000 mutations in >400 genes. Analysis of the TILLING data revealed that EMS is a nearly ideal mutagen, producing G/C-to-A/T transitions >99% of the time with only minor local sequence biases [17]. Our analysis also indicated that the high-throughput cleavage-based detection method is highly efficient: at least 3/4 of all mutations present were detected and essentially all mutations detected were confirmed by sequencing.

ATP demonstrated the practicality of high-throughput TILLING in a production setting. However, the small genome size of Arabidopsis and the ease with which it can be cultured might have made Arabidopsis easier to TILL than a field-grown crop plant with a large genome. To determine whether the procedures that were developed for ATP can be generalized, we chose to TILL maize, a crop plant with a genome that is ~20 times larger than Arabidopsis. We find that essentially the same procedures that provided efficient TILLING of Arabidopsis can be applied to maize, yielding comparable results. Despite the relatively small size of our mutagenized maize TILLING population, we obtained useful mutations. These include a promising allelic series for a chromomethylase gene that had been previously implicated in non-CpG DNA methylation, whose counterpart in Arabidopsis is responsible for epigenetic gene silencing and genome surveillance.

## Results

**Figure 1:**
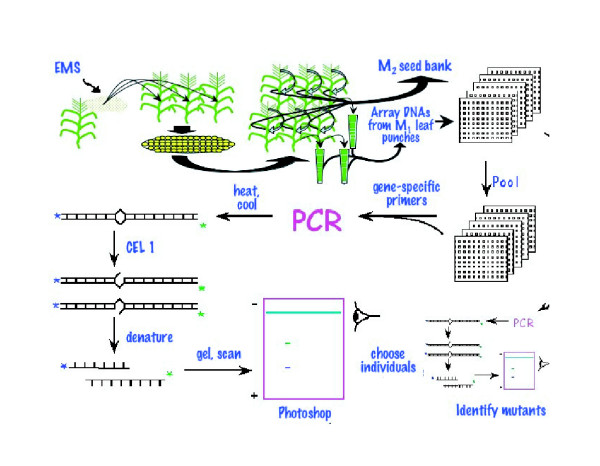
**Schematic diagram of maize TILLING. **Fresh pollen is collected and mutagenized with ethylmethanesulfonate (EMS). Pollen is then applied to silks of wild-type plants from the same genetic background. Seeds from the resulting ears are grown into plants of the M1 generation. Plants of this generation are heterozygous for any induced mutation. Tissue is collected either from each M1 plant or from approx. 10 M2 siblings from the M1 self cross. M3 seed is generated by randomly intermating 10–12 M2 siblings. This M3 seed serves as the seed stock for future studies. DNA is extracted from collected tissue and samples are pooled to increase screening throughput. For mutation detection, sequence specific primers are used to amplify the target locus by PCR. Following amplification, samples are heat denatured and reannealed to generate heteroduplexes between mutant amplicons and their wild-type counterparts. Heteroduplexes are cleaved using CEL I endonuclease and are visualized using denaturing polyacrylamide gel electrophoresis. See reference [34] for further details.

TILLING consists of a series of steps, beginning with chemical mutagenesis of reference individuals and culminating in the determination of mutant base pairs by DNA sequencing [14]. High-throughput TILLING utilizes the CEL I mismatch-cleavage enzyme on heteroduplexes with detection of end-labeled cleavage products on electrophoretic gels [15,18]. The procedure for maize TILLING is identical to that for Arabidopsis, except that pollen rather than seed was treated with EMS (Figure 1).

For this study, two separate mutagenized B73 maize populations, designated UI (M2 families from 384 mutagenized lines) and NS (366 individual M1 plants), were screened in 4-fold pools (4 UI families or 4 NS plants) in a 96-well format. To compensate for the larger genome size of maize relative to Arabidopsis, we increased the amount of genomic template DNA amplified 20-fold; otherwise, all protocols and default parameters were the same as used for Arabidopsis TILLING [16]. We found that 11 of the 14 primers gave high quality products, compared to ~90% success that is obtained for Arabidopsis. We proceeded to screen for mutations within these 11 gene fragments in the 750 DNA samples and discovered 21 point lesions (Table 1). All 21 were verified by sequencing.

Of the 21 lesions, 17 appeared to be EMS-induced. These 17 mutations were G/C-to-A/T mutations, as expected for EMS [17]. The other four lesions were found in a single plant (Table 1) and only one was a G/C-to-A/T transition, suggesting that this plant is a non-B73 contaminant, likely due to cross-pollination. Contaminations seen as an excessively high frequency of polymorphisms in single plants have been occasionally observed by ATP [17,19]. The presumed EMS-induced mutations were detected in single plants, except for DMT102 G878A, where the exact same mutation was found in two different plants; this circumstance is expected and observed to occur 4% of the time in Arabidopsis based on random distribution of induced mutations, GC content of the genome and distribution of location of mutations discovered in fragments [17]. Finding one coincidence among 17 mutations is not significantly different from expectation. Furthermore, finding that all 17 mutations are G/C-to-A/T transitions effectively rules out the possibility that they are naturally occurring polymorphisms: with four possible single-base changes, the chance probability of observing that all 17 conform to the expectation for EMS mutagenesis is only (1/4)^17 ^or ~1/10^10^.

Importantly, each population yielded 8–9 confirmed new mutations, for an overall mutation density of approximately two mutations/megabase. Taking into account the fact that pollen treatment mutagenizes only one of two genomes, whereas seed treatment mutagenizes both (although by screening M2s in the latter case, 1/4 of the mutations are lost as +/+ segregants), the estimated mutation density for both maize populations is ~3/4 as high as our average for Arabidopsis per mutagenized genome.

We have previously demonstrated reliable detection of mutations in 8-fold pools based on analysis of ATP-generated data [17]. For example, we obtained almost precisely the expected 2:1 heterozygote:homozygote ratio for ~1900 mutations in 8-fold pools, indicating that detection of 1/16 is no different from detection of 1/8 by TILLING. To confirm this detection efficiency in maize, we screened the primer sets for DMT101 and DMT103 with 8-fold pools from the 750 B73 DNA samples that had already been screened in pools of four. In this test, we detected only three of the five mutations that we had discovered by 4-fold pooling. Inadequate data quality does not account for missing these two base changes (UI20291 and NS3471.9) in 8-fold pools, because the gel images were typical of what is seen in our ATP operation. Therefore, we considered the possibility that failure to detect two of five mutations in this limited test was caused by variation of DNA amounts in the pools.

One potential source of variability in DNA amount is that degraded DNA is difficult to measure accurately when visualized by agarose gel analysis. We noticed that some of the genomic DNA samples, including NS3471.9, were partially degraded. Inaccuracies in measuring the amount of DNA in a sample will compromise normalization in pools: any variation in the amount of DNA contributed by each plant in the pool will lead to reduced representation of one or more plants. As the amount of DNA in a pool from a particular plant decreases, mutation detection becomes limiting. Recognizing that the quality of genomic DNA could potentially hinder the throughput of mutation detection, we sought an alternative method of sample quantification and normalization. We have found that running samples on 3% Metaphor^® ^(Cambrex) agarose gels reduced "smearing" of fragments, thus facilitating quantification.

While lower DNA quality and inaccurate normalization could account for missing the base change in NS3471.9, this is not a likely explanation for missing the mutation in UI20291, which was from an apparently undegraded sample. Therefore, we considered that another source of sample-to-sample variation arose from the sampling of leaves from M2 sibling plants in the UI series rather than using M1 plants directly. Individual DNA samples from the UI population were generated by pooling approximately 10 individuals from an M2 family. M1 plants that are heterozygous for a new mutation will yield M2 families segregating 1:2:1 for the new mutation. A sample over a large M2 family should yield DNA that is equal parts wild type and mutant allele, assuming good viability and fertility of the mutant allele. However, a small M2 sample may be biased and contain too large a proportion of wild-type DNA which would then prevent a given target sequence containing a mutation from being detected among 8-fold pooled DNAs, depending on the limit for robust mutation detection.

Direct evidence that sampling from M2 families was a problem came from our finding that two of the mutations found in plants sampled as leaves from multiple M2 plants were scored as homozygous by our usual criteria. Pollen mutagenesis can only produce M1 heterozygotes, and we interpret the homogeneity of the mutant as resulting from limited sampling of heterogeneous M2 plants. For example, if only one or two of the planted M2 seed germinated for these families, and the limited sample favored homozygotes, then they would sometimes appear to be purely homozygous in the sequence trace. Similarly, an equal or greater number of mutations will be underrepresented because wild type will be in excess, leading us to miss detecting the mutation in 8-fold, but not in 4-fold pools. This underscores the importance of using DNA from M1 pollen-mutagenized individuals and taking care to avoid collection procedures that could exacerbate degradation during DNA isolation. By assaying DNA concentrations on Metaphor gels and sampling only the M1 generation, we should be able to pool 8-fold without reducting detection efficiency. To test this, we screened a population of 768 M1 W22 maize DNAs (pollen mutagenesis) with similar degradation patterns as the B73 samples described above and normalized using 3% Metaphor agarose gels. We then made 8-fold and 4-fold pools from these 768 samples and screened with four of the 11 primer sets in the original screen. This screening of 6-Mb of total sequence led to the independent detection of the same 3 mutations in both the 4-fold and the 8-pools (data not shown). Therefore, we conclude that even partially degraded DNA from M1 pollen-mutagenized samples, such as might be extracted from material collected in the field, can be used for TILLING.

**Figure 2:**
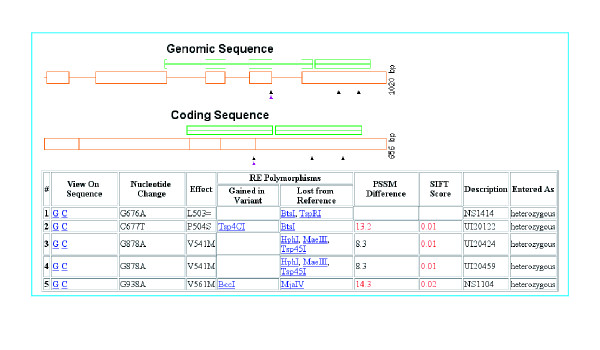
**PARSESNP output for maize DMT102. **At top is a map showing the positions of five independent mutations in the TILLed fragment, based on the gene model (red boxes for exons and lines for introns) and block alignments produced by running SIFT with DMT102 as query against SWISS-PROT-Trembl. The table provides information concerning the effect of the mutation and restriction site changes that can be used for genotyping progeny plants. PSSM Difference or SIFT Scores in red indicate that the missense mutation is predicted to damage the protein. PARSESNP also provides sequence maps (not shown).

The 17 induced mutations discovered in the screen were distributed as expected, consisting of 10 missense, 7 silent and no truncation mutations, compared with 51% missense, 44% silent and 5% truncation mutations based on ~4000 TILLed Arabidopsis mutations. Considering that about half of missense mutations are expected to be damaging to a typical protein [20], we expect that even a small allelic series will be useful for phenotypic analysis. Indeed, we discovered that all three different DMT102 missense mutations are likely to be deleterious to the protein, based on SIFT and PSSM Difference scores (Figure 2). The SIFT algorithm predicts deleterious missense mutations with ~75% overall accuracy based on analysis of experimental mutagenesis data [21,22] and comprehensive human polymorphism and disease data [23]. Therefore, the DMT102 allelic series appears to be essentially complete after screening a 1-kb region within only 750 maize plants.

## Discussion

We have shown that TILLING is an efficient method for reverse genetics in a crop plant. The density of mutations that we discovered appears to be only slightly lower than what is obtained for Arabidopsis using the same methodology. For Arabidopsis, we currently screen ~2300 M2 plants to obtain a suitable allelic series, which averages ~12 mutations per 1.5-kb segment screened. Based on this work, we estimate that screening ~4000 maize plants will provide a comparable series ([4000] [17 mutations] [1.5-kb/1-kb]÷([11-kb] [750 plants]) = ~12 mutations). Since this study was completed, we have expanded the size of our mutagenized population to ~2500 M1 individuals in the B73 genetic background and ~2000 M1 plants in the W22 genetic background that are ready for screening, and populations of ~10,000 M1 plants are being prepared.

For effective pooling, each individual in a pool must be represented at a concentration that is equivalent to the other members in a pool. Failure to accomplish this could result in a mutation represented in the pool below the level of detection. The goal is to maximize throughput by increasing pooling while still detecting all possible mutations. We have shown that in Arabidopsis, heterozygous mutations can be as efficiently discovered as homozygous mutations in 8-fold pools, thus providing a minimum estimate of robust discovery in a production setting of 1 in 16 [16]. We have described here two possible sources of error that might hinder the ability to pool samples effectively: inaccurate DNA quantitation, and sampling error in tissue collection. DNA quantitation using gel electrophoresis is difficult when samples are degraded, as the standard band of DNA appears as a smear, although the problem can be minimized using high percentage agarose gels. Sampling bias in the collection of individuals descended from a single mutagenized parent can also lead to non-equivalent representation of a mutation in a pool. The present study revealed normalization inaccuracy or sampling bias by detecting homozygous mutations in lines that should have yielded only heterozygous mutations. To minimize sampling bias, only DNA from M1 individuals will be used to create our library for a maize TILLING service.

At least one of the maize genes that we screened yielded an excellent allelic series. We discovered three missense mutations in DMT102, all of which are predicted to damage the protein based on sequence conservation. This analysis used two different programs: SIFT (Sorting Intolerant From Tolerant [23], which uses PSI-BLAST alignments, and PARSESNP Project Aligned Related Sequences and Evaluate SNPs[24], which provides a PSSM (Position-Specific Scoring Matrix) Difference score based on alignment blocks (Figure 2). DMT102 is a member of the chromodomain-containing "chromomethylase" subfamily of cytosine-5-DNA methyltransferases. The Arabidopsis CMT3 chromomethylase is the first example of a gene to be TILLed [14], and a nonsense mutation was responsible for sharply reducing CpNpG methylation [25]. This had confirmed a study in which a Mutator insertional mutation into maize DMT102 was shown to reduce CpNpG methylation [26]. Plant chromomethylases have received considerable recent attention. For example, studies of other mutations affecting CpNpG methylation reveal the first links between DNA methylation, histone methylation [27] and the small interfering RNA (siRNA) machinery [28] in a higher eukaryote. A methylation profiling study has revealed that transposons are *in vivo* targets of CMT3-dependent methylation [29]. Together with the Mutator insertional mutation [26], our TILLed DMT102 allelic series may now be applied to understanding the relationship between DNA methylation, chromatin structure, siRNAs and transposon biology in maize.

## Conclusions

TILLING has several advantages as a general reverse-genetic tool, especially for organisms for which other options are limited. The high density of mutations resulting from chemical mutagenesis means that, relative to insertional or deletional mutagenesis, far fewer plants are required for screening and much smaller genes can be effectively targeted. EMS is a stable and reliable mutagen, whereas the stability, penetrance and accuracy of RNAi-based silencing is uncertain [30,31], and insertional mutagenesis can cause chromosomal rearrangements that complicate subsequent phenotypic analysis [32]. TILLING provides an allelic series of mutations, and is the only method that can focus the search for missense mutations to just part of a protein, such as in a single domain of a multidomain protein. TILLING lines can be produced in a homogeneous wild-type genetic background, which avoids problems of heterogeneity often required for insertional mutagenesis, especially in maize. Finally, given the high regulatory and intellectual property costs associated with transgenics and the current concerns about genetically modified crop plants, there is likely to be agricultural interest in producing phenotypic variants without introducing foreign DNA of any type into a plant's genome. We are currently establishing the reference population necessary to provide TILLING as a service to the maize community.

## Methods

### Maize mutagenesis, culture and DNA preparation

B73 pollen was mutagenized with EMS and applied to the silks of B73 ears [33]. Ears were harvested at 5–6 weeks post pollination. For the NS population, M1 seed were planted and a whole young leaf harvested from each plant and lyophilized for DNA sampling. For the UI population, the M1 were selfed to make M2 seed, then families of twenty M2 siblings were planted and a total of 60 leaf discs were punched from the youngest leaves using all members of the family, and the pooled sample for an entire family lyophilized.

Samples were prepared from lyophilized leaf tissue essentially as described [34], except that dried tissue was homogenized into a powder in a FastPrep homogenizer before adding buffer, and 20 mg of this powder was used to prepare DNA.

### High-throughput TILLING

The same procedure used for TILLING Arabidopsis [16] was adapted for maize with only minor modifications. Primers were designed to amplify ~1-kb segments using the CODDLE program [35] based on either known maize genomic sequence or from maize cDNAs that are orthologous to intronless rice sequence. Genes were chosen from the NSF Plant Chromatin Project web site [36] based on the availability of genomic sequence or on the prediction of an exonic region at least 1-kb in size. Because rice and maize typically have identical placement of introns, by aligning the predicted maize coding sequence with the rice gene model, we could choose maize-specific primers that would amplify only exonic DNA. To find such regions, cDNA sequences were searched against Arabidopsis and rice genomic sequences using a version of BLAST that was modified to identify large exonic regions in maize based on the corresponding regions being exonic in rice and/or Arabidopsis. In all, we were able to design 14 primer pairs from plant chromatin genes for screening, and primer sets were ordered. In other cases, maize genomic sequence was available from ChromDb. Amplification of pools and individual DNA samples in 96-well plates, annealing, cleavage by CEL I, electrophoresis, image analysis, rescreening and DNA sequencing were performed as described [16].

Screening of mutations using LI-COR gel analyzers was performed as previously described [34]. Sequence trace information was analyzed using the Sequencher program as described [17]

## Authors' contributions

SHR, KY and EB prepared the samples, CB, CAC, LCE and ARO performed the high-throughput screening and data processing, CW and NS provided the mutagenized plant material, EAG performed the data analysis and BJT, LC and SH participated in the design and execution of the study and wrote the paper.

## Figures and Tables

**Table 1:** Mutations detected by screening EMS-mutagenized maize populations.

Gene^1^	Individual	Sequence	Stock Number
DMT101	3D3	C553T	UI20118
	8A8	G464A	UI20291
DMT102	3H3	C677T	UI20122
	11B3	G878A	UI20424
	12B3	G878A	UI20459
	15G3	G938A	NS2809.1
	19C2	G676A	NS3457.3
	21G2	A637W	NS3471.9^2^
DMT103	4A9	G116A	UI20152
	14E9	G514A	NS2806.7
	16G9	G519A	NS2810.4
	21G2	G120K	NS3471.9^2^
	21G2	A148G	NS3471.9^2^
	21G2	G789A	NS3471.9^2^
DMT106	15E2	C697T	NS2808.6
HAC110	16B7	G471A	NS2809.12
HDA105	4A3	G343A	UI20152
	9F2	C571T	UI20333
	13H2	G271A	NS2804.1
	22A9	G417A	NS3478.7

^1^No mutations were detected in 1 kb fragments of five other genes screened: DMT105, SDG104, SDG105, SDG124 and SDG125. ^2^Multiple non-G/C-to-A/T mutations discovered in NS3471.9 indicate that it derives from contaminating pollen. See text for details.
